# E-Beam Cross-Linking of Complex Hydrogels Formulation: The Influence of Poly(Ethylene Oxide) Concentration on the Hydrogel Properties

**DOI:** 10.3390/gels8010027

**Published:** 2021-12-31

**Authors:** Maria Demeter, Ion Călina, Anca Scărișoreanu, Marin Micutz

**Affiliations:** 1 Accelerators Laboratory, National Institute for Lasers Plasma and Radiation Physics, 077125 Măgurele, Romania; calina.cosmin@inflpr.ro (I.C.); anca.scarisoreanu@inflpr.ro (A.S.); 2 Department of Physical Chemistry, University of Bucharest, 030018 Bucharest, Romania; micutz@gw-chimie.math.unibuc.ro

**Keywords:** hydrogels, e-beam, cross-linking, rheology, collagen, PEO

## Abstract

In the present study, we report on the complex hydrogels formulations based on collagen-poly(vinyl pyrrolidone) (PVP)-poly(ethylene oxide) (PEO) cross-linked by e-beam irradiation in an aqueous polymeric solution, aiming to investigate the influence of different PEO concentrations on the hydrogel properties. The hydrogel networks’ structure and their composition were investigated using equilibrium swelling degree, complex rheological analysis, and FT-IR spectroscopy. Rheological analysis was performed to determine the elastic (G′) and viscous (G″) moduli, the average molecular weight between cross-linking points (Mc), cross-link density (V_e_), and the mesh size (ξ). The effect of the PEO concentration on the properties of the hydrogel was investigated as well. Depending on the PEO concentration added in their composition, the hydrogels swelling degree depends on the absorbed dose, being lower at low PEO concentrations. All hydrogel formulations showed higher G′ values (9.8 kPa) compared to G″ values (0.2 kPa), which shows that the hydrogels have a predominantly elastic behavior. They presented stability greater than 72 h in physiological pH buffers and reached equilibrium after 25 h. The Mc parameter is strongly dependent on the PEO concentration and the absorbed dose for all hydrogel compositions. The cross-linking density increased with the absorbed dose.

## 1. Introduction

To be classified as a superabsorbent hydrogel, the newly developed material has to absorb considerable amounts of water, saline solution, or physiological fluids as high as 10–1000 times their weight, owing to the considerable number of hydrophilic groups contained in their structure [1]. Because of their unique properties, superabsorbent hydrogels are suitable for use as wound dressings, disposable diapers, or as scaffolds for tissue engineering and as a drug delivery release matrix [2]. Superabsorbent hydrogels designed for wound dressings applications are typically obtained by mixing the collagen with other water-soluble polymers and chemical cross-linking agents, to increase the hydrogel properties according to their targeted specifications [3,4,5,6,7,8].

Collagen is the main protein component for most soft tissues and has the property of maintaining the structural integrity of tissues. These qualities recommend it to be the most used biopolymer for the production of biomaterials [9].

From the investigations in the literature, we found that the method of cross-linking with ionizing radiations that involve γ-radiation was more intensively used to obtain hydrogels, compared to e-beam cross-linking.

The use of e-beam radiation processing, which generally produces very pure materials, is environmentally friendly and the processes are economical [10,11,12,13,14]. Another convenience of the radiation processing method is the ability to choose the structure and properties of hydrogels obtained from the same components, by a suitable choice of processing parameters [15,16,17].

Regarding the use of e-beam as a method of cross-linking and simultaneous sterilization, it has been used since 1991 [18]. Subsequently, e-beams were used for hydrogels cross-linking made from various polymers singles like PVP [19], PEO [20] or different mixtures compositions [21]. 

In our first studies aimed at the synthesis and characterization of superabsorbent hydrogels based on collagen and PVP, the hydrogels were synthesized by γ-radiation, in the absence of oxygen and without the addition of chemical cross-linking agents. Hydrogels showed a swelling degree value up to 2000% and a high gel fraction above 90%. In addition, the sol-gel analysis shows that the cross-linking process predominates over the degradation process [22].

Subsequently, another study was published in which collagen/PVP-based hydrogels without and with cross-linking agents, were synthesized with e-beams. In addition, the experimental results showed that the swelling ratio, the gel fraction, and elastic modulus showed optimal values for one of the hydrogels prepared in the presence of 0.5% cross-linking agents and cross-linked with 7.5–10–12.5 kGy [23].

Another previous study presented the properties of collagen/PVP/PAA/PEO quaternary hydrogel which was synthesized by e-beam cross-linking. The cross-linking of the hydrogel takes place simultaneously with its sterilization, due to the established radiation doses. The hydrogel obtained has properties that recommend it to be used for dressings, controlled drug delivery systems, and as a matrix in soft tissue engineering [24].

Regarding the polymer mixtures used in the present study based on collagen-polyvinylpyrrolidone (PVP) and different concentrations of polyethylene oxide (PEO), no study was found in the literature related to the e-beam cross-linking method.

In the present study, we describe the e-beam cross-linking of collagen-polyvinylpyrrolidone (PVP) mixed with different concentrations of polyethylene oxide (PEO) to obtain superabsorbent hydrogels. The effect of absorbed dose and the PEO concentration on hydrogels properties was investigated. Sol-gel analysis was used to determine gelation dose, correlated with radiation chemical cross-linking yields. The hydrogel network structure and their composition were investigated using equilibrium swelling degree in simulated physiological conditions or a case of an infected wound, rheological analysis, and FT-IR spectroscopy. Rheological analysis was performed to determine the elastic (G′) and viscous (G″) moduli, the average molecular weight between cross-linking points (Mc), cross-link density (V_e_), and the mesh size (ξ). 

## 2. Results and Discussion

### 2.1. Sol-Gel Analysis

**Figure 1:**
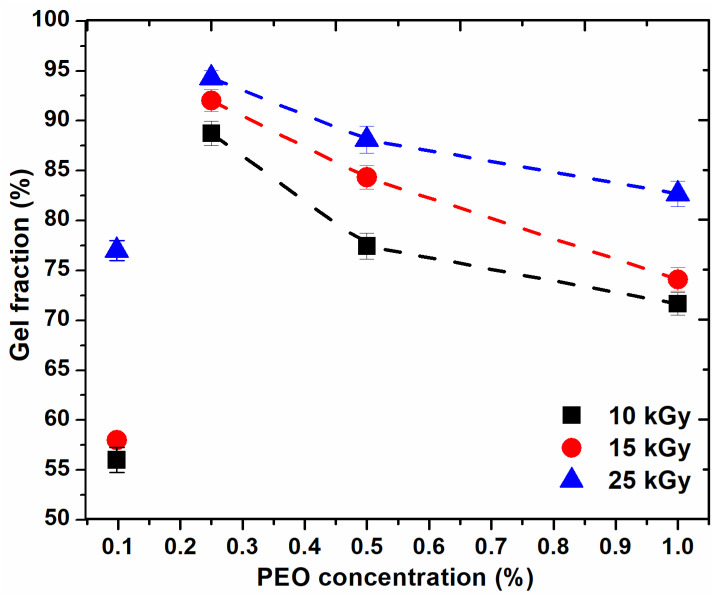
Variation of the gel fraction depending on the PEO concentration.

Figure 1 shows the variation of the gel fraction (GF%) as a function of PEO concentration and absorbed dose. The figure shows that GF (%) increases with an increase in the absorbed dose and decreases with an increase in PEO concentration.

For the hydrogel composition prepared with 0.1% PEO, GF (%) is below 60% for doses lower than sterilization dose. For the systems prepared with PEO (0.25–0.5%), GF (%) are higher than 75% and for those systems prepared with PEO (0.5–1%), GF (%) decreases, but not significantly, up to 72%. From the above curves, it appears that, for any dose, the recommended concentration range is between (0.2–0.5)%. The higher the concentration of polymers, the higher the viscosity of the polymer system; thus the reduced mobility of the macroradicals resulting from irradiation. Increasing the concentration of PEO in these systems above 0.5% does not favor the cross-linking processes, also at concentrations below 0.2% PEO, cross-linking does not predominate.

Table 1 and Table 2 show the results that describe the irradiation process with different PEO concentrations.

According to the experimental data presented in Table 1 and Table 2, it was observed that at the lowest concentration of 0.1% PEO, sol-gel analysis data cannot be estimated. This might be due to the higher scissions process within this polymeric blend, as was demonstrated by the radiochemical yields of degradation. At the concentration of 1% PEO, the gelling dose is low, and the p_0_/q_0_ parameters decrease with the concentration having the lowest value at the concentration of 0.25% PEO, which highlights an appreciable cross-linking. In systems prepared with (0.25–0.5)% PEO, G(X) and G(S) increase with dose and G(X) > G(S), which again highlights the predominance of cross-linking processes, to the detriment of polymers degradation. In systems prepared with (0.1% PEO, G(S) > G(X) highlights the predominance of degradation processes.

Additionally, it is observed that at a minimum and maximum PEO concentration, the cross-linking yields are almost equal and are twofold lower than the other compositions, being slightly higher only at the dose of 25 kGy. For hydrogels prepared with 0.25% PEO, the values of G(S) are about 6 times lower than those of G(X), which shows that the crosslinking process predominates. At lower and highest PEO concentration the degradation yields either exceed or are equal with the G(X) values, showing a predominance of scissions process within hydrogels network structure.

In our previous study, we showed the obtaining of collagen/PVP/PAA/PEO quaternary hydrogels prepared with 0.3% PEO in an inert atmosphere of argon, where G(X) ≈ G(S) [24]. In this study, it follows that successive centrifugation → degassing → vacuum operations and using an optimal PEO concentration is sufficient to obtain higher quality hydrogels with G(X) >> G(S), with stable and homogeneous network structure.

According to literature data for several irradiated polymeric materials at room temperature and in the absence of oxygen, the radiochemical yields of cross-linking, G(X) and degradation, G(S) have the following values (Table 3) [25].

From the literature, it appears that materials with low values of G(X) and G(S), respectively, are irradiation-resistant, for example, styrene. Usually, polymers containing several hydrogen atoms located on the sides of the chains (i.e., polyethylene) tend to cross-link under the action of ionizing radiation. Polymers that contain methyl groups or halogen atoms in their molecules tend to degrade. Polymers containing aromatic nuclei in their molecule (i.e., polystyrene) are usually stable on irradiation.

The following cases are encountered in practice: when G(X) >> G(S), the molecular weight increases continuously due to continuously cross-linked bonds; however, the molecular weight at some point will not increase, because G(S) will increase faster. In the case when G(X) ≥ G(S), G(S) at the end of the reaction will equal G(X) and the molecular weight will register a turning point, and the general reaction will result from cross-linking to degradation. When G(X) << G(S), degradation occurs continuously [25].

### 2.2. Rheological Analysis

**Figure 2:**
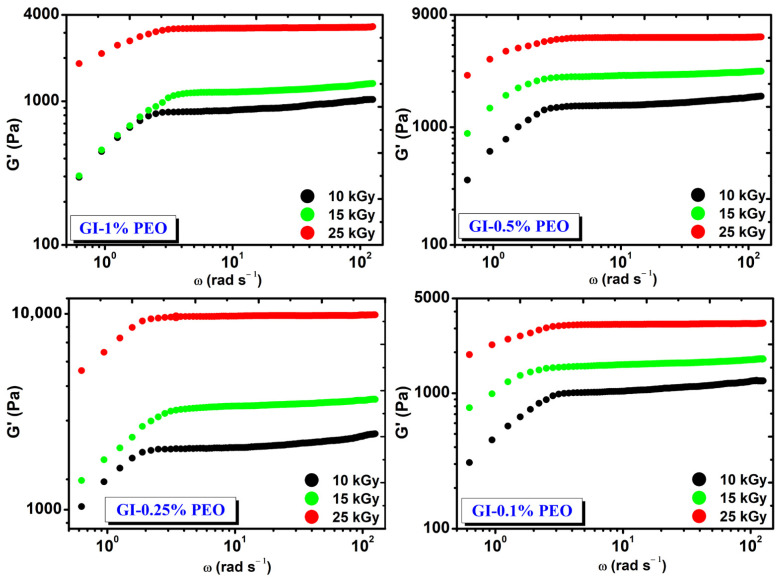
The variation of G′ depending on the angular frequency (ω) and the absorbed dose.

Figure 2 shows the rheological behavior of hydrogels prepared with various concentrations of PEO at absorbed doses of (10–25) kGy. Regardless of the added concentration of PEO in each system, the G′ modulus increased with the dose and is constant in the investigated angular frequency range.

At the absorbed dose of 25 kGy, the modulus G′ had values between (3241–9816) Pa and depended on the PEO concentration, having the highest value at 0.25% PEO. 

**Figure 3:**
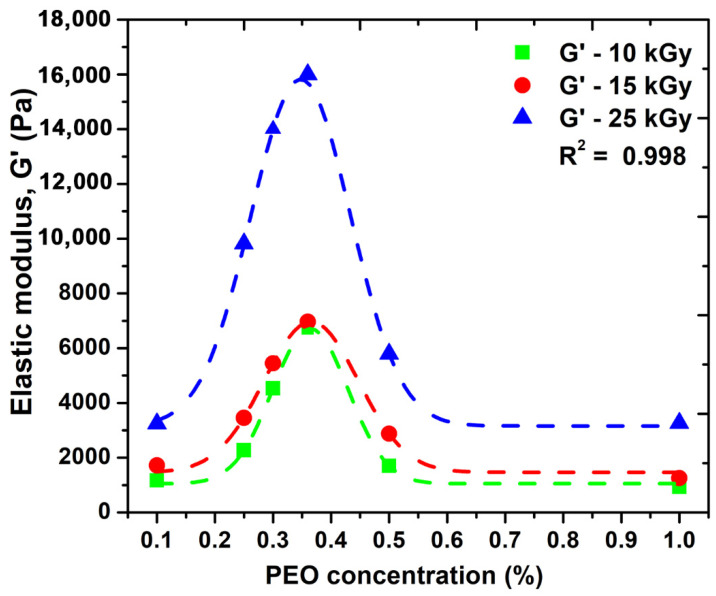
The effect of PEO concentration on the elastic modulus, G′.

Figure 3 shows the variation of the elastic modulus (G′) as a function of PEO concentration.

We observe that the elastic modulus increases and depending on the absorbed dose; however, after a certain concentration of PEO is introduced into the system, there is an advanced decrease in this parameter. Here, as can be seen from the graph, a concentration of 0.35% PEO could be considered optimal for the significant improvement of the elastic properties of such systems. Moreover, the possibility of cross-linking this system without the addition of chemical cross-linking agents can be considered. A higher PEO concentration above 0.35% within hydrogel network structure could decrease the overall crystallinity of such systems, which could be associated with destabilizing the hydrogel network, making it more brittle, and consequently decrease the elastic properties. Another explanation could be related with the distribution of PEO molecules within hydrogel network up to a given concentration produces an increase in the network mesh size as a consequence of effective reduction of total crosslink concentration. This process is responsible for the reduction in the hydrogels elastic moduli and is in good agreement with the network studies presented in the Section 2.4. Similar behavior has been observed for PVA/water systems additives with 2% PEO [26].

**Figure 4:**
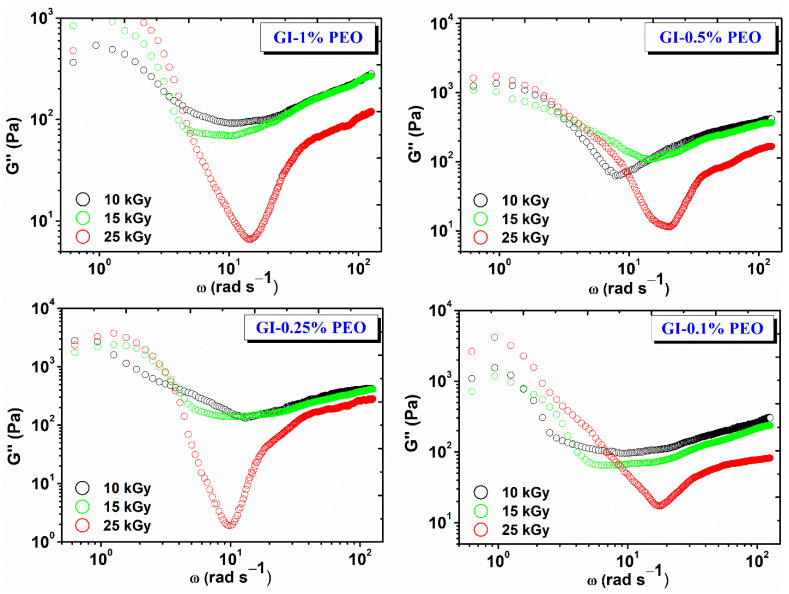
Variation of G″ depending on the angular frequency (ω) and the absorbed dose.

Figure 4 shows the variation of the viscous modulus (G″) depending on the angular frequency (ω) and the absorbed dose. For each hydrogel composition, G″ decreases with dose, which shows that the cross-linking density and elastic contribution is favored with increasing dose, and the viscous behavior is inhibited [27].

The nonlinear evolution of G″ at low frequencies suggests the onset of a structural change in the hydrogel network, which most likely has a viscous behavior. This is the case with cross-linked gels, when the experimental interval in which the stress is applied becomes comparable to the relaxation time of the gel—in this case, at lower test frequencies.

The G′/G″ ratio plays an important role in the application of gels in various fields, i.e., which is recommended to be less than 50. For example, in the literature, it has been reported that, for some gels obtained from gelatin and enzymes, the G′/G″ ratio > 50, these gels can be recommended as biomaterials, as they do not dissociate in contact with various wounds [28]. For the investigated systems, the G′/G″ ratio, values were between 38–49; the highest values were found for the hydrogels prepared with (0.25–0.5)% PEO at 25 kGy.

For all samples over the whole frequency range studied, G′ is higher than G″, which shows that hydrogels have predominantly elastic behavior specific to solid materials with elastic properties. This also indicates that the lifetime of reversible bonds in the system is longer than the time chosen to perform the experiment [29].

### 2.3. The Swelling Degree and Hydrogels Stability in Simulated Physiological Environments

**Figure 5:**
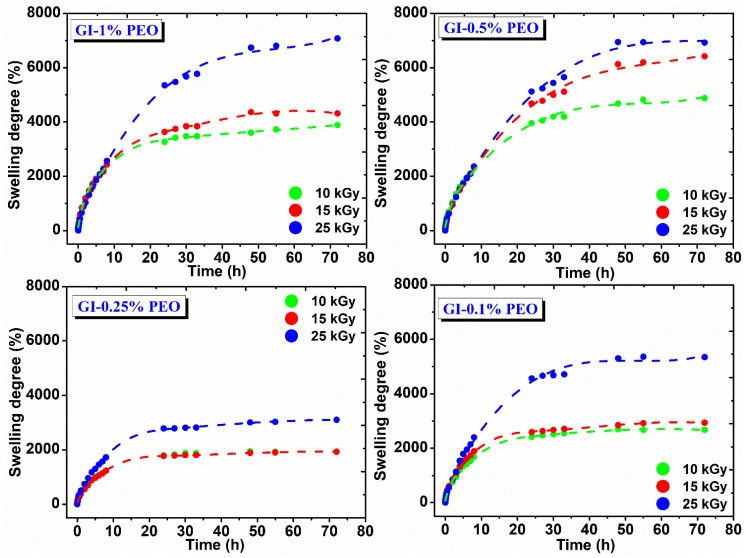
Swelling degree in DI water for hydrogels prepared with (0.1–1)% PEO.

Figure 5 shows the swelling degree variation in DI water at 37 °C as a function of immersion time and the absorbed dose. In the body environment or on a wound site, hydrogels may have a different answer, which influences their final swelling, network structure, permeability, and mechanical properties [30]. 

Thus, the pH change from acidic to basic, characteristic behavior of an infected wound stage can dramatically influence the general properties of the hydrogel. The hydrogels have different charges, depending on chemical groups within their network. For this reason, they are classified as neutral, ionic, and amphoteric. The swelling capacity of ionic hydrogels is directly influenced by their chemical composition and the pH variations of the surrounding medium. Usually, acidic and basic groups ionize to form a stable network, in this way are developed fixed charges, which turn on in electrostatic repulsions, solvent penetration in hydrogel network, and swelling. Anionic hydrogels swell better at higher pH due to the deprotonation (pH > pK_a_ of ionizable groups). In contrast to this, cationic hydrogels at low pH swell due to the ionization of pendant amine groups [31].

The swelling degree of hydrogels prepared with various PEO concentrations depends on the absorbed dose; their composition increases with the absorbed dose and decreases with PEO concentration, either high or low. For example, hydrogels prepared with (0.5–1)% PEO showed a swelling degree between 3000% and 7100%, which means that these hydrogels can incorporate into their structure an amount of ~30 to ~70 g water/g hydrogel.

For hydrogels prepared with 0.25%, respectively 0.1% PEO, the swelling degree is between 1500% to 5500%, which means that these gels contain a reduced amount of water, of ~15 to 55 g water/g hydrogel. These hydrogels showed stability for 72 h in the swelling medium and reached equilibrium after 25 h, having a maximum absorption 8 h after immersion in the swelling medium, being able to absorb on average 25 g water/g hydrogel.

Another important detail was that hydrogels prepared at lower and higher PEO concentration exhibited a lower cross-link density, correlated with low mechanical strength, therefore a large amount of water can be absorbed within hydrogel network. The hydrogels prepared with 0.25% PEO showed a higher elastic modulus and cross-link density and the lowest hydrogel mesh sizes, which explains the considerable reduction in its swelling capacity.

**Figure 6:**
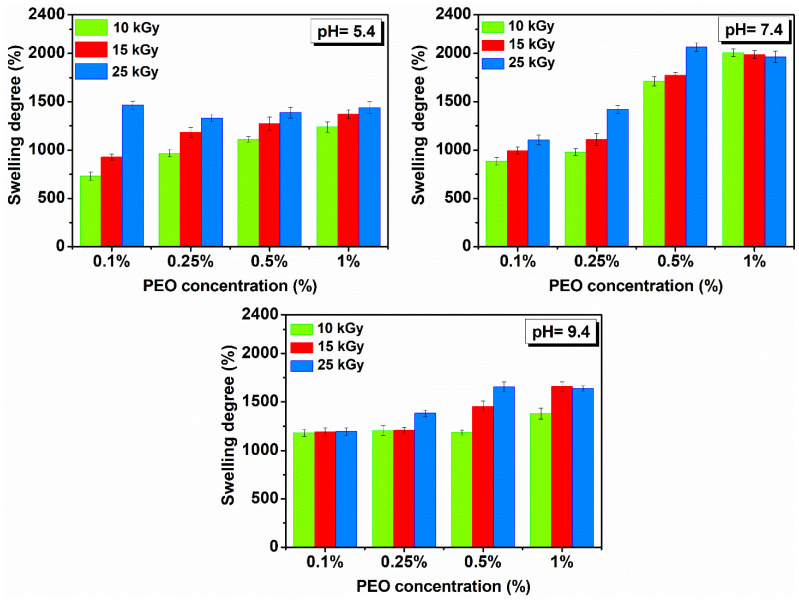
Swelling in solutions with pH = 5.4–9.4 and temperature of 37 °C determined 72 h for hydrogels prepared with (0.1–1)% PEO.

Figure 6 shows the hydrogels swelling degree prepared with (0.1–1)% PEO in solutions with different pH.

It is observed that SD (%) is more reduced compared to SD (%) determined in DI water and is in all cases higher under neutral pH conditions. Under different pH conditions, the investigated hydrogels showed stability of over 48 h and reached equilibrium after 8–10 h. All hydrogels have a liquid absorption capacity in the pH range from 5.4 to 9.4 of ~10 to 20 g water/g hydrogel.

Swelling is favorable for the hydrogel produced with 25 kGy. If the concentration of PEO is further reduced, the swelling is higher in a neutral and weakly basic environment. Here, the hydrogels reach equilibrium much faster, compared to other systems, i.e., less than 6 h, in a weakly acidic and neutral environment.

For a general assessment of the quality of the prepared hydrogels another important parameter which describes hydrogel swelling is percentage equilibrium water content (EWC%) [32].

**Figure 7:**
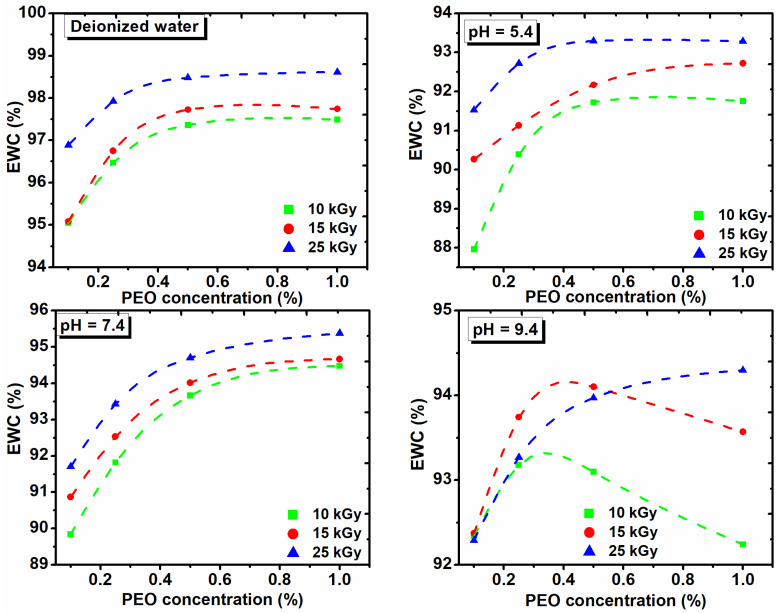
The effect of PEO concentration on the equilibrium water content in deionized water and solutions with different pH and absorbed dose.

The EWC% as function on the PEO concentration, both in DI water and in solutions of various pHs and with the absorbed dose is shown in Figure 7. These hydrogels show low EWC% at acidic pH, while the EWC% increases as the pH of the medium increases.

Figure 7 shows that the percentage of EWC in DI water increases with the dose and concentration of PEO. At pH of 5.4 and 7.4, the EWC% increases with the dose in the range (10–15) kGy and with the concentration of PEO. At pH of 9.4, the swelling increases with the concentration of PEO for the dose of 25 kGy. The EWC% of hydrogels cross-linked with 10 kGy and 15 kGy, increases up to 0.35% PEO, after which it decreased drastically. The presence of ionizable groups such as anionic groups in a hydrogel with a dense network, are deprotonated in basic pH, leading to a more hydrophilic hydrogel with a higher absorption capacity. We demonstrate that collagen/PVP/PEO hydrogels show important swelling properties in different pH environments and absorbed dose. Moreover, it was demonstrated that the PEO concentration critically influenced the swelling properties.

### 2.4. Characterization of Hydrogels Network Structure

Table 4 shows the values of the structural parameters of the hydrogels. The parameters M_C_, V_e_, and ξ were determined according to the data obtained from the rheological analysis using Equations (7)–(9).

The parameter M_C_ (the molecular weight between two successive cross-linking points) decreased with increasing radiation dose for compositions (0.25–1)% PEO. For all doses, in the concentration range (0.25–1)% PEO, M_C_ decreased with a decrease in concentration. The cross-linking density (V_e_), increased with the irradiation dose, being maximum at 25 kGy for the prepared system with 0.25% PEO. The parameter ξ decreased with the irradiation dose for all concentrations. The parameters ρ, $\nu_{2,r}$ and $\nu_{2,s}\;$ were determined using an analytical balance equipped with a density kit and the results were used to determine the structural parameters (M_C_, V_e_, and ξ) presented above.

### 2.5. FT-IR Analysis

**Figure 8:**
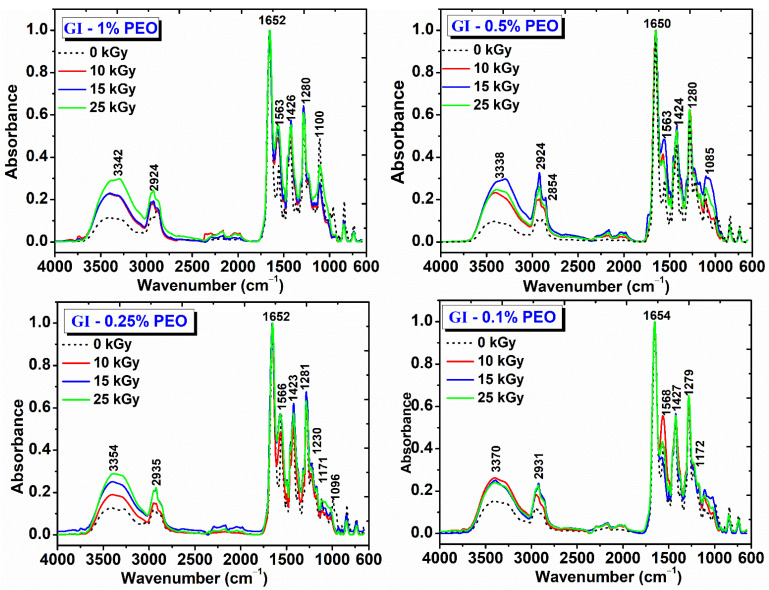
FTIR spectra for hydrogels prepared with (0.1–1)% PEO.

The obtained hydrogels were evaluated by FT-IR spectroscopy. Figure 8 shows the FT-IR spectra for hydrogels prepared with various concentrations of PEO and doses of (0–25) kGy. The spectra obtained are interpreted in relation to the spectra of the non-irradiated polymer mixtures and according to those of the single component polymers [33,34,35].

The main peaks identified for the non-irradiated GI-1% PEO hydrogel are: wide absorption band in the region (3700–2700) cm^−1^, with a maximum absorption at 3373 cm^−1^, stretching vibrations specific to OH and NH groups; (2883–2950) cm^−1^, stretching vibrations specific to CH_2_ and CH bonds; 1654 cm^−1^ (amide I) and 1556 cm^−1^ (amide II), peaks characteristic of the collagen molecule; 1427 cm^−1^, deformation vibrations specific to CH_2_ groups in the pyrrolidine ring; 1282 cm^−1^, stretching vibrations of the amide III band; 1224 cm^−1^, stretching vibration specific to the C−O group; 1109 cm^−1^, stretching vibration of the −C−O−C− group, characteristic to the PEO structure.

The GI-1% PEO hydrogels cross-linked with doses of (10–25) kGy show wide absorption bands in the region (3700–2700) cm^−1^; the bands intensities increased with the absorbed dose, and the peak maximum shifted to smaller wavenumbers, up to 3354 cm^−1^. The CH_2_ peaks shifted to smaller wavenumbers, up to 2924 cm^−1^, while the amide I bands remains unchanged. The amide II band was shifted slightly toward higher wavenumbers and increased in intensity, from 0.4 (0 kGy) to 0.7 (25 kGy). The band at 1107 cm^−1^ decreased slightly in intensity.

The non-irradiated GI-0.5% PEO hydrogel shows a wide absorption band with maximum absorption at 3384 cm^−1^ (OH and NH group); 2885 cm^−1^ and 2950 cm^−1^ (stretching vibrations specific to CH, CH_2,_ and CH_3_ bonds); 1655 cm^−1^ (amide I), and 1558 cm^−1^ (amide II); 1430 cm^−1^ (deformation vibrations specific to CH_2_ groups in the pyrrolidine ring); 1281 cm^−1^ (stretching vibrations of the amide band III); 1222 cm^−1^ (stretching vibration specific to the C-O group); 1109 cm^−1^ (stretching vibration of the −C−O−C− group).

The GI—0.5% PEO hydrogels cross-linked with doses of (10–25) kGy show that at the doses of 10 kGy and 25 kGy the intensity of the absorption bands decreases, and at the dose of 15 kGy the intensity of the absorption bands increases. The peak assigned to amide I band remains unchanged and the peaks from 3346 cm^−1^ (10 kGy) shifted to 3370 cm^−1^ (25 kGy). As the irradiation dose increased, the peak assigned to the amide band II shifted from 1563 cm^−1^ to 1574 cm^−1^ and decreased in intensity. The peak attributed to the −C−O−C− groups shifted from 1110 cm^−1^ to 1105 cm^−1^, probably due to strong molecular associations between the participating polymers.

The non-irradiated GI-0.25% PEO hydrogel are: wide absorption band with a maximum absorption at 3370 cm^−1^ (OH and NH group); the other characteristic peaks were identical to the spectra described for GI hydrogels prepared with (0.5–1)% PEO. The C-OH peak decreased in intensity. The GI—0.25% PEO hydrogels cross-linked with doses of (10–25) kGy, which shows that the increase in the absorbed dose, increases the intensity of the bands in the range (3700–2700) cm^−1^, and the peak maximum shifted to 3354 cm^−1^. The position of the other peaks remained unchanged after irradiation with 10 and 15 kGy.

The non-irradiated GI-0.1% PEO hydrogel are: wide absorption band with a maximum absorption at 3368 cm^−1^ (OH and NH group); the other characteristic peaks were identical to the spectra described for GI hydrogels prepared with (0.5–1)% PEO. For the GI—0.1% PEO hydrogels cross-linked with doses of (10–25) kGy, the intensity of the characteristic peaks was not affected in the region (3700–2700) cm^−1^ of the spectrum.

As general evaluation after FTIR analysis of this complex hydrogel’s formulation, it can be pointed out that, even at 25 kGy, the characteristic peaks of the collagen molecule, the molecule known to be very sensitive to the action of ionizing radiation, can be easily identified. Moreover, the characteristic peak of all constituents’ polymers could be identified after e-beam irradiation, which suggests maintaining of the structural integrity of hydrogels. 

The shifting towards higher wavenumbers of the main peaks and the broadening of the peaks situated in the region 3700–2700 cm^−1^ could be associated with the cross-linking reaction between collagen and PEO molecules. The increasing of relative band intensities, as a consequence of higher absorbed dose, suggests the formation of intermolecular interactions through H-bonds between collagen molecules, PVP and PEO, which demonstrates the chemical interaction of these polymers following of e-beam cross-linking [34,36]. 

## 3. Conclusions

Collagen/PVP/PEO hydrogels with elastic and superabsorbent properties were prepared by e-beam cross-linking. The effect of PEO concentration on collagen/PVP mixtures was studied. The increase in the PEO concentration in these systems over 0.5% does not favor the cross-linking processes; at concentrations below 0.2% PEO, the cross-linking does not predominate.

In the case of systems prepared with (0.25–0.5)% PEO, the values of G(X) are higher than those of G(S) and are doubled at the maximum of absorbed dose. Compared to hydrogels prepared with 0.3% PEO in an inert atmosphere of Ar, the values G(X) ≈ G(S) showed that cross-linking and degradation occurs simultaneously, which suggests that these hydrogels formulation can be produced without including complicated operations in the synthesis process. Degassing of the system through successive operations of centrifugation, degassing and vacuuming, are sufficient operations to obtain a hydrogel with controlled properties by irradiation.

The G′ modulus increased with the absorbed dose and was also constant in the investigated angular frequency range. The G′ modulus at 25 kGy had values between (3241–9816) Pa and depended on the PEO concentration, having the highest value at 0.25% PEO. From the evaluation of the elastic modulus (G′) variation depending on the concentration of PEO, it was observed that a concentration of 0.35% PEO could be considered optimal, for the significant improvement of the elastic properties of such systems, for the possibility cross-linking of the collagen/PVP/PEO system, without the addition of chemical cross-linking agents and without special conditions, such as cross-linking in an inert atmosphere. All hydrogels showed high values of G′ compared to G″ values, which shows that the hydrogels have a predominantly elastic behavior specific to solid materials with elastic properties.

The swelling degree of hydrogels depends on the absorbed dose and the composition of hydrogels, increases with the absorbed dose, but decreases at high or low concentrations of PEO. Depending on the PEO concentration added in the composition of hydrogels, the swelling degree varied between (1500–5500–7100%), which means that these hydrogels can absorb ~(15–70) g water/g hydrogel. 

They presented stability greater than 72 h, reaching equilibrium after 25 h. The swelling degree is reduced under lower pH conditions and is maximum at neutral and basic pH. Under pH conditions (5.4–9.4), all hydrogels showed stability over 48 h and have an absorption capacity of ~(10–20) g water/g hydrogel.

The M_C_ parameter depends on the PEO concentration and the absorbed dose for all hydrogel compositions. The cross-linking density increased with the absorbed dose.

## 4. Materials and Methods

### 4.1. Materials

Type I acidic collagen was provided by the Collagen Department from Leather and Footwear Research Institute. Poly(N-vinyl pyrrolidone) (Mw = 360,000 g/mol), poly (ethylene oxide) (Mw = 300,000 g/mol), acrylic acid (Mw = 72.06 g/mol), sodium hydroxide (99% purity) and N’N-Methylene bis(acrylamide) (Mw = 154.17 g/mol) were purchased from Merck and without further purifications.

### 4.2. Hydrogel Fabrication

The polymeric solutions were prepared according to the procedure presented in our previous work [24], and the composition of each mixture is shown in Table 5.

After complete homogenization of the collagen/PVP mixture, it was divided into 4 equal parts and (0.1–1)% PEO was added.

**Figure 9:**
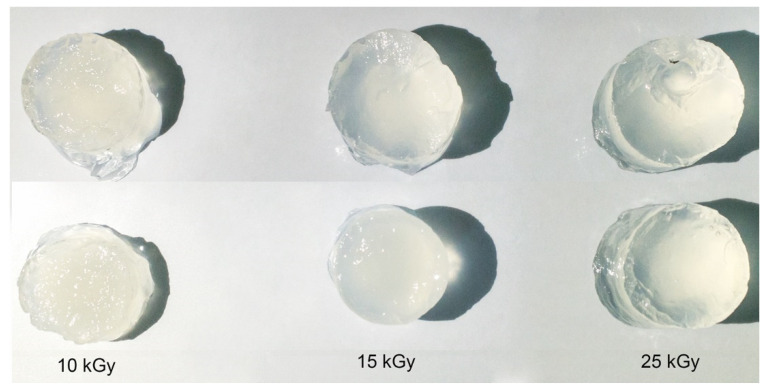
Digital photographs of the cross-linked hydrogels at 10, 15 and 25 kGy.

The irradiation of the polymer solutions in order to obtain the hydrogels by crosslinking with e-beam was performed in the Electron Accelerator Laboratory at the linear electron accelerator (ALID 7, 6 MeV), owned by INFLPR. The e-beam dosimetry was performed following the procedure, according to ISO/ASTM 51631:2020 standards using graphite calorimeters [37]. Before cross-linking with e-beam, the same operations (centrifugation → degassing → vacuum) were performed to remove dissolved oxygen introduced during mixing operations to reduce oxidative processes during irradiation. These operations ensured that a hydrogel with a uniform surface and without gas bubbles is obtained. Figure 9 shows images with hydrogels obtained after e-beam cross-linking.

### 4.3. Sol-Gel Analysis

The e-beam cross-linked hydrogels were cut into small pieces (~2 g) and vacuum-dried at 40 °C until a constant weight (W_i_) was achieved, then immersed in DI water (48 h at room temperature). After 48 h, the hydrogels were taken out and dried at 40 °C to a constant weight (W_d_). The gel fraction (GF) and the sol fraction (s) were calculated using equations:

(1) $$\mathrm{GF}\left(\%\right)=\left(\frac{W_{d}}{W_{i}}\right)$$

(2) s=1−GF

All measurements were calculated as the average of three determinations for each sample.

The gelation dose and the degradation vs. cross-linking ratios for the hydrogels were determined with Gelsol95 software, which was based on the Charlesby–Rosiak formula [38]:

(3) $$s+\sqrt{s}=\frac{p_{0}}{q_{0}}+\left(2-\frac{p_{0}}{q_{0}}\right)\left(\frac{D_{v}+D_{g}}{D_{v}+D}\right)$$

where p_0_ is the degradation density (i.e., average number of main chain scissions per monomer unit and per unit dose), q_0_ is the cross-linking density (i.e., fraction of monomer units cross-linked per unit dose), D (kGy) is the absorbed dose, D_g_ (kGy) is the gelation dose, and D_V_ (kGy) is the virtual dose (the dose necessary to transform the real sample into a sample with the molecular weight distribution of M_w_/M_n_ = 2).

The radiation yields of cross-linking (G(X)) and degradation (G(S)) were calculated using the following equations [39]:

(4) $$G\left(X\right)=\frac{4.9\cdot 10^{2}\cdot c}{M_{C}\cdot D\cdot \rho }$$

(5) $$G\left(S\right)=G\left(X\right)\cdot 2\frac{p_{0}}{q_{0}}$$

where G(X) is expressed as the number of moles of cross-linking bonds per Joule, G(S) is the radiation yield of chain scission (mol/J), M_C_ (kg/mol) is the average molecular weight between two successive cross-links, c (g/L) is the polymer concentration in the irradiated solution, D (J/kg) is the absorbed dose, and ρ (kg/m^3^) is the polymer density.

### 4.4. Rheological Analysis

The dynamic rheological parameters of storage modulus (G′) and loss modulus (G″) were determined to evaluate the stability of the hydrogel network 

Dynamic rheological measurements of the hydrogels were performed by employing a micro Fourier rheometer MFR 2100 (GBC, Australia) equipped with a homemade temperature control jacket connected to a circulating water bath Lauda E100.

The operating parameters of the instrument during the rheological investigation were as follows: the gap between plates—400 μm; displacement amplitude—0.03 μm (to fall into the linear viscoelasticity domain); frequency domain—0.005–2.000 Hz (with a step of 0.005 Hz, which led to angular frequencies, in rad/s, of 2π times higher than the corresponding frequencies taken in Hz); equilibration time for each isothermal measurements—20 min, and 30 scans per rheogram. All the measurements were conducted in triplicate at the same constant temperature of 23 °C and all values were expressed as mean values and standard deviations.

### 4.5. The Degree of Swelling and the Stability of Hydrogels in Simulated Physiological Environments

The degree of swelling and the stability of hydrogels in simulated physiological environments were determined both in deionized water and in solutions of different pH’s (5.4, 7.4, and 9.4) at physiological temperature (37 °C) until equilibrium condition was reached. The degree of swelling was calculated as a function of the dry (W_d_) and swollen (W_s_) hydrogel weights using the following equation [40]

(6) $$\mathrm{SD}\left(\%\right)=\frac{\left(W_{s}-W_{d}\right)}{W_{d}}\cdot 100$$

Citric acid–Na_2_HPO_4_ buffer solution, pH of 5.4, was prepared from 21.01 g/L citric acid monohydrate, C_6_H_8_O_7_ H_2_O, 35.61 g/L Na_2_HPO_4_ 2H_2_O in 1000 mL water.

Phosphate buffered saline (PBS, pH = 7.4) was prepared from 8 g NaCl, 0.2 g KCl, 1.44 g Na_2_PO_4_, 0.24 g KH_2_PO_4_ in 1000 mL DI water.

Sodium carbonate–sodium bicarbonate buffer solutions, pH of 9.4 was prepared using 10.599 g/L Na_2_CO_3_ anhydrous and 8.4 g/L NaHCO_3_ in 1000 mL DI water. The pH buffer solutions were prepared according to the recommendations of Sigma-Aldrich (Merck, Darmstadt, Germany) regarding the biological buffers guide.

### 4.6. Characterization of Hydrogels Network Structure

The network parameters as the molecular weight between two successive cross-linking points (M_C_), cross-linking density (V_e_) and mesh size (ξ) were calculated using the values of the elastic modulus (G′) determined from rheological experiments and based on the rubber elasticity theory, using the following equations [41,42,43,44]:

(7) $$M_{c}=\frac{A\rho \mathrm{RT}{\left(\nu_{2,r}\right)}^{2/3}{\left(\nu_{2,s}\right)}^{1/3}}{G^{\prime }}$$

(8) ξ=ν2,s−13·[Cn(2McMr)]−12·l

(9) $$V_{e}=\frac{\rho }{M_{c}}$$

(10) $$\nu_{2,r\left(s\right)}=\frac{{\left[1+\left(m_{2r\left(s\right)}-1\right)\cdot \rho_{\mathrm{hydrogel}}\right]}^{-1}}{\rho_{\mathrm{solvent}}}$$

where ρ (kg/m^3^) is the polymer density, R is the universal gas constant (8.314 m^3^ Pa/molK), T is the absolute experimental temperature (298.15 °K), $\nu_{2,r}$ is the polymer volume fraction after e-beam cross-linking, $\nu_{2,s}$ is the polymer volume fraction of the cross-linked hydrogel in swollen state, l is the carbon–carbon bond length (0.154 nm), and the factor A equals 1 for an affine network and 1–2/ϕ for a phantom network. C_n_ was taken as a weighted average of the characteristic ratios of the polymers: collagen = 9 [45]; PVP = 12.3 [46]; PEO = 4 [47] and AA = 6.7 [48]. $M_{r}$ is the monomeric unit of collagen, PVP, PEO and AA, taken as an average (collagen = 321.32 g/mol; PVP = 112.88 g/mol; PEO = 44.05 g/mol and AA = 72.06 g/mol). The polymer volume fractions $\nu_{2,r}$ and $\nu_{2,s}$ were determined using Equation (10).

### 4.7. FT-IR Analysis

The FT-IR spectra of unirradiated and irradiated samples were taken with a Perkin Elmer, Spectrum 100 instrument equipped with a diamond crystal. The hydrogels samples were dried to constant mass and used for FT-IR analysis. The FT-IR spectra were acquired in ATR mode in the wavelength range of 4000 to 600 cm^−1^ with 50 scans/sample and a resolution of 4 cm^−^^1^.

## Figures and Tables

**Table 1:** Sol-gel analysis for hydrogels with different concentrations of PEO.

Hydrogel Code	$p_{0}/q_{0}$	$D_{g}(\mathrm{kGy})$	R^2^
GI—1% PEO	0.41	0.08	0.93
GI—0.5% PEO	0.37	5.21	1.00
GI—0.25% PEO	0.09	4.28	0.93
GI—0.1% PEO	-	-	-

**Table 2:** Radiochemical yields of cross-linking G(X) and degradation G(S).

Hydrogel Code	G(X) μmol J^−1^			G(S) μmol J^−1^			G(S)/G(X) (25 kGy)
	Absorbed Dose (kGy)						
	10	15	25	10	15	25	
GI—1% PEO	0.22	0.20	0.36	0.18	0.17	0.30	0.83
GI—0.5% PEO	0.41	0.48	0.65	0.30	0.36	0.48	0.74
GI—0.25% PEO	0.42	0.45	0.84	0.07	0.08	0.15	0.17
GI—0.1% PEO	0.26	0.26	0.34	0.37	0.37	0.48	1.41

**Table 3:** Values from the literature data for radiochemical yields G(X) and G(S) for various polymeric materials.

Polymer Material	G(X)	G(S)	G(S):G(X)
Low density polyethylene	1.42	0.48	0.34
High density polyethylene	0.96	0.19	0.20
Polymethylmethacrylate (PMMA)	<0.5	1.1–1.7	>2
Natural rubber	1.3–3.5	0.1–0.2	0.14
Polyvinyl acetate	0.1–0.3	0.06	0.2
Polymethylacrylate	0.45–0.52	0.08	0.15
Polystyrene	0.019	0.0094	0.4

**Table 4:** Experimental values for G′, G″, M_C_, V_e_, ξ and ρ.

**Hydrogel GI-1% PEO**								
**Dose** **(kGy)**	${\bar{G}}^{\prime }$	${\bar{G}}^{\prime \prime }$	$M_{C}$	$V_{e}$	**ξ**	ρ	$\left(\nu_{2,r}\right)$ **^2/3^**	$\left(\nu_{2,s}\right)$ **^1/3^**
	**(Pa)**	**(Pa)**	**(kg mol^−1^)**	**(mol m^−3^)**	**(nm)**	**(kg m^−3^)**		
10	937	187	228.54	0.45	72	1033	0.2419	0.3454
15	1253	184	163.95	0.62	63	1028	0.2412	0.3338
25	3260	85	54.98	1.87	42	1028	0.2432	0.2889
**Hydrogel GI-0.5% PEO**								
**Dose** **(kGy)**	${\bar{G}}^{\prime }$	${\bar{G}}^{\prime \prime }$	$M_{C}$	$V_{e}$	**ξ**	ρ	$\left(\nu_{2,r}\right)$ **^2/3^**	$\left(\nu_{2,s}\right)$ **^1/3^**
	**(Pa)**	**(Pa)**	**(kg mol** **^−1^)**	**(mol m** **^−3^)**	**(nm)**	**(kg m** **^−3^)**		
10	1700	300	122.18	0.85	58	1046	0.2573	0.3113
15	2876	270	68.98	1.51	46	1037	0.2591	0.2978
25	5790	117	30.82	3.36	34	1034	0.2593	0.2685
**Hydrogel GI-0.25% PEO**								
**Dose** **(kGy)**	${\bar{G}}^{\prime }$	${\bar{G}}^{\prime \prime }$	$M_{C}$	$V_{e}$	**ξ**	ρ	$\left(\nu_{2,r}\right)$ **^2/3^**	$\left(\nu_{2,s}\right)$ **^1/3^**
	**(Pa)**	**(Pa)**	**(kg mol** **^−1^)**	**(mol m** **^−3^)**	**(nm)**	**(kg m** **^−3^)**		
10	2267	332	119.64	0.86	45	1033	0.2691	0.3936
15	3452	309	73.83	1.40	38	1036	0.2671	0.3715
25	9816	207	23.73	4.36	24	1035	0.2678	0.3390
**Hydrogel GI-0.1% PEO**								
**Dose** **(kGy)**	${\bar{G}}^{\prime }$	${\bar{G}}^{\prime \prime }$	$M_{C}$	$V_{e}$	**ξ**	ρ	$\left(\nu_{2,r}\right)$ **^2/3^**	$\left(\nu_{2,s}\right)$ **^1/3^**
	**(Pa)**	**(Pa)**	**(kg mol** **^−1^)**	**(mol m** **^−3^)**	**(nm)**	**(kg m** **^−3^)**		
10	1163	206	191.74	0.53	63	1025	0.2450	0.3583
15	1716	164	127.00	0.81	53	1032	0.2460	0.3459
25	3241	82	58.25	1.77	42	1035	0.2475	0.2972

**Table 5:** Composition of hydrogels with various concentrations of poly (ethylene oxide) (PEO).

Hydrogel Sample	PVP (%)	Collagen (%)	PEO (%)	AA (%)	NaOH (%)	NMBA (%)	H_2_O (%)
GI—1% PEO	7	0.3	1	2.8	1.3	0.5	87.1
GI—0.5% PEO	7	0.3	0.5	2.8	1.3	0.5	87.6
GI—0.25% PEO	7	0.3	0.25	2.8	1.3	0.5	87.9
GI—0.1% PEO	7	0.3	0.1	2.8	1.3	0.5	88.1
